# An α-tocopheryl succinate enzyme-based nanoassembly for cancer imaging and therapy

**DOI:** 10.1080/10717544.2018.1446476

**Published:** 2018-03-08

**Authors:** Song Yi Lee, Hyun-Jong Cho

**Affiliations:** College of Pharmacy, Kangwon National University, Chuncheon, Republic of Korea

**Keywords:** α-tocopherol succinate, curcumin, lysozyme, nanoassembly, tumor targeting

## Abstract

Nanoassembly (NA) based on a D-α-tocopherol succinate (αTS) conjugated lysozyme (Lys) (Lys-αTS) was fabricated for tumor-selective delivery of curcumin (CUR) for breast cancer therapy. Lys and αTS were used as a biocompatible enzyme and a hydrophobic residue, respectively, for the preparation of nanocarriers in this study. Compared with CUR-loaded cross-linked Lys (c-Lys/CUR) NA, Lys-αTS/CUR NA exhibited a smaller hydrodynamic size (213 nm mean diameter), a narrower size distribution, and a more spherical shape. Sustained drug release was observed from the Lys-αTS/CUR NA for five days at a normal physiological pH (pH 7.4). The developed Lys-αTS/CUR NA showed enhanced cellular accumulation, antiproliferative effects, and apoptotic efficacies in MDA-MB-231 human breast adenocarcinoma cells. According to the results of optical imaging test in the MDA-MB-231 tumor-bearing mouse models, the Lys-αTS/CUR NA-injected group exhibited a more tumor-selective accumulation pattern, rather than being distributed in the normal tissues and organs. The observed tumor targetability of Lys-αTS/CUR was further studied, which revealed improved *in vivo* anticancer activities (better inhibition of tumor growth and induction of apoptosis in the tumor tissue) after an intravenous administration in the MDA-MB-231 tumor-bearing mouse models. All these results indicate that the newly developed enzyme-based nanocarrier, the Lys-αTS NA, can be a promising candidate for the therapy of breast cancers.

## Introduction

Recently, a number of formulation strategies have been developed for cancer therapy (Yoon et al., 2015; Kemp et al., 2016; Kim et al., 2016; Tran et al., 2016; Song et al., 2017). Because of their toxicity-related properties, the tumor-selective delivery of anticancer agents is a crucial issue in the development of formulations for anticancer drugs. It is known that nanocarriers are capable of tumor-specific drug delivery based on the enhanced permeability and retention (EPR) effect, which may be considered as a passive tumor targeting strategy (Matsumura & Maeda, 1986; Maeda et al., 2000). To compensate for the drawbacks of passive tumor targeting based on the EPR effect, various active tumor targeting strategies (such as the introduction of tumor-targeting ligands to nanocarrier development) have been designed (Danhier et al., 2010; Lammers et al., 2012; Shim et al., 2017). Although remarkable anticancer activities of diverse nanocarriers have been verified in cell culture and animal models, only a few of them have been approved for clinical use. One of the major obstacles in extending the application of the developed nanocarriers to human systems is the toxicity of the pharmaceutical excipients comprising the nanocarriers. To overcome these toxicities, biocompatibility and biodegradability have been regarded as important requisites during the development of pharmaceutical formulations. Synthetic polymers [such as poly(lactic-co-glycolic acid) and polycaprolactone] and natural polymers (such as hyaluronic acid) are approved by the US Food and Drug Administration (FDA) for clinical applications (Danhier et al., 2012; Li & Tan, 2014; Zhang et al., 2014).

With the use of these polymers, intrinsic proteins (such as the human serum albumin [HSA]) have been also widely studied for the preparation of nanoformulations (Hawkins et al., 2008; Elsadek & Kratz, 2012). HSA is the most abundant protein in the plasma (35–50 g/L in human serum) and therefore it has been used for the development of injection dosage forms (Elzoghby et al., 2012; Zhang et al., 2016; Liu et al., 2017). HSA nanoparticles can be prepared by several preparation methods, such as coacervation, emulsification, thermal gelation, nanospray drying, nanoparticle albumin-bound technology, & self-assembly (Elzoghby et al., 2012). The albumin-bound paclitaxel (nab-paclitaxel) nanoparticle has been approved by the USFDA in 2005 and is available in the markets (Abraxane, Abraxis BioScience; Gradishar, 2006; Elsadek & Kratz, 2012). It reduces the immunological responses that can be induced by foreign materials contained in polymeric nanocarriers (Elzoghby et al., 2012). A number of modified HSA-based nanoparticles have been developed for anticancer drug delivery to the tumor region (Byeon et al., 2016; Liu et al., 2017).

Similar to albumin-based nanocarriers, several lysozyme (Lys) nanovehicles have been developed for the delivery of drug cargos (Li et al., 2015; Lin et al., 2015; Mahanta et al., 2015). Lys has 129 amino acids with a molecular weight of 14.4 kDa (Canfield, 1963). Chemically, it is known as *N*-acetylmuramide glycanhydrolase and it can hydrolyze the 1,4-beta-linkages between the *N*-acetylmuramic acid and the *N*-acetyl-D-glucosamine residues of peptidoglycans. Lys is abundant in milk, mucus, saliva, and tears and it is recognized as a part of the immune system. Its antibacterial and anticancer activities have also been elucidated in recent years (Aminlari et al., 2014; Mahanta et al., 2015). Self-assembled Lys nanogels, alone or in combination with other materials, have been developed as drug delivery systems (Li et al., 2015; Lin et al., 2015). In this study, D-α-tocopherol succinate (αTS) was chemically conjugated to Lys as a hydrophobic residue. αTS was shown to inhibit cancer cell proliferation while inducing apoptosis in cancer cells via mitochondrial destabilization, without affecting the proliferation of normal cells (Prasad et al., 2003). Moreover, it can also enhance the inhibition of tumor growth as in case of ionizing radiation and hyperthermia therapy (Prasad et al., 2003; Angulo-Molina et al., 2014). Because of the anticancer activities of αTS and its derivatives, they have been used as a component of nanoparticles for cancer therapy (Zeng et al., 2013; Tao et al., 2015; Zeng et al., 2015; Mallick et al., 2016; Muddineti et al., 2017; Palao-Suay et al., 2017). Herein, αTS-conjugated Lys (Lys-αTS) was synthesized and curcumin (CUR) was incorporated into the Lys-αTS nanoassembly (NA). The physicochemical properties, *in vitro* anticancer activities, *in vivo* tumor targetability, and the *in vivo* anticancer activities of the Lys-αTS/CUR NA were assessed.

## Materials and methods

### Materials

CUR, deuterium oxide (D_2_O), hexadeuterodimethyl sulfoxide (DMSO-d_6_), Lys (from chicken egg white, ∼70000 U/mg), *N*-hydroxysuccinimide (NHS), *N*-(3-dimethylaminopropyl)-*N*'-ethylcarbodiimide hydrochloride (EDC), and poly(ethylene glycol) 400 (PEG 400) were purchased from Sigma-Aldrich (Saint Louis, MO). Sodium dodecyl sulfate (SDS), αTS, and the 2,4,6-trinitrobenzene sulfonic acid (TNBS) assay kit were obtained from Tokyo Chemical Industry Co., Ltd. (Tokyo, Japan). Cy5.5-NHS was purchased from BioActs (DKC Corp., Incheon, Korea). Roswell Park Memorial Institute (RPMI) 1640 medium, penicillin, streptomycin, and heat-inactivated fetal bovine serum (FBS) were acquired from Gibco Life Technologies, Inc. (Grand Island, NY). All the other reagents were of analytical grade and purchased from commercial sources.

### Synthesis and characterizations of c-Lys and Lys-αTS

To fabricate the structure of the Lys-based NA, αTS was covalently bonded as a hydrophobic residue to Lys. To synthesize the Lys-αTS, an EDC/NHS-coupled amide bond was formed between the carboxylic acid group of αTS and the amine group of Lys. αTS (21.2 mg, 0.04 mmol) was dissolved in dimethyl sulfoxide (DMSO, 12 mL) and the pH was adjusted to a value of 4.0 by adding 1 N HCl. EDC (11.5 mg, 0.06 mmol) and NHS (6.7 mg, 0.06 mmol) were dissolved in that solution by stirring and the pH value was adjusted to 7 by adding 1 N NaOH. Lys (288 mg, 0.02 mmol) dissolved in DMSO (12 mL) was slowly added to the αTS/EDC/NHS solution and the mixture was stirred for 24 h. The solution of the mixture was then dialyzed against distilled water (DW) for two days with a dialysis membrane (molecular weight cutoff [MWCO]: 6–8 kDa). The resultant was freeze-dried for future use, after adding sucrose (1%, w/v). As a control group, cross-linked Lys (c-Lys) was synthesized via an EDC/NHS-coupled reaction. c-Lys was synthesized by the same procedure, without the addition of αTS.

The synthesis of c-Lys and Lys-αTS was evaluated by sodium dodecyl sulfate-polyacrylamide gel electrophoresis (SDS-PAGE). According to Laemmli’s discontinuous method (Laemmli, 1970), gel electrophoresis was performed with a running gel (20% acrylamide) and a stacking gel (5% acrylamide). Lys, c-Lys, and Lys-αTS were blended with the Laemmli sample buffer and were heated at 95 °C for 5 min to denature the proteins. A 20 µL aliquot of the sample and a protein marker (EzWay Protein-Multicolor Ladder, KOMA Biotech, Seoul, Korea) were loaded into the wells. The loaded amount of Lys, c-Lys, and Lys-αTS was 10 µg. The electrophoresis was carried out for 90 min at 100 V. The gel was then stained with Coomassive R250 (Bio-Rad Laboratories, Inc., Hercules, CA) for 30 min and then was de-stained overnight.

Lys, c-Lys, and Lys-αTS were dispersed in the phosphate buffered saline (PBS, 5 mM, pH 7.2) using a Lys concentration of 0.1 mg/mL. They were analyzed by the Chirascan^TM^-plus circular dichroism (CD) spectrometer (Applied Photophysics Ltd., Surrey, UK). The path length (1 mm) was fixed within the quartz cell. The step size was 1 nm, the bandwidth was 1 nm, and the wavelength ranged between 200 and 260 nm.

The fluorescence spectra of Lys, c-Lys, and Lys-αTS were obtained by using a fluorescence spectrometer (FP-6500, Jasco Corp., Tokyo, Japan). Lys, c-Lys, and Lys-αTS were dispersed in PBS (5 mM, pH 7.2) at a Lys concentration of 0.1 mg/mL. Each emission spectrum (300–500 nm) was scanned at a fixed excitation wavelength of 280 nm.

The amine groups of Lys, c-Lys, and Lys-αTS were quantitatively determined by the TNBS assay. Lys, c-Lys, or Lys-αTS was dissolved in the reaction buffer (0.1 M sodium bicarbonate, pH 8.5) at 50 μg/mL. A 0.25 mL aliquot of the TNBS solution (0.01%, w/v) was added to each of the protein solutions (0.5 mL). The mixtures were incubated at 37 °C for 2 h. SDS (10%, w/v) solution (0.25 mL) and 1 N HCl (0.125 mL) were then added to each of the mixtures. The absorbance was measured at 335 nm with a multi-mode microplate reader (SpectraMax i3, Molecular Devices, Sunnyvale, CA), and the relative absorbance was calculated by comparing the absorbance of each group to that of the Lys group.

The molecular weights of c-Lys and Lys-αTS were assessed by matrix-assisted laser desorption/ionization time-of-flight (MALDI-TOF) mass spectrometry. The matrix used was 2,5-dihydroxybenzoic acid (DHB), and the Voyager DE-STR mass spectrometer (Applied Biosystems, Framingham, MA) was used for all the analyses. The instrument was operated in a linear mode with nitrogen lasers (337 nm) and the accelerating voltage was 25 kV.

The particle characteristics of c-Lys and Lys-αTS dispersion in DW were assessed by a dynamic light scattering (DLS) method (ELS-Z1000; Otsuka Electronics, Tokyo, Japan) according to manufacturer’s protocol. c-Lys or Lys-αTS was dispersed in DW (5 mg/mL) by vortex-mixing for 5 min and their mean diameters and polydispersity index values were also measured.

### Fabrication and characterizations of CUR-loaded NAs

CUR was encapsulated as a hydrophobic model drug in the Lys-αTS NAs by the dialysis method (Jeong et al., 2017). Both CUR (6 mg) and Lys-αTS (96 mg) were dissolved in DMSO (6 mL). The solution was then transferred to a dialysis bag (MWCO: 6–8 kDa) and was dialyzed against DW for 6 h. The resultant was lyophilized after adding sucrose (1%, w/v). In case of the c-Lys/CUR NA, 96 mg of c-Lys was used instead of Lys-αTS for preparing the c-Lys/CUR NA using the same procedure.

The CUR contents in the c-Lys/CUR NA and the Lys-αTS/CUR NA were quantitatively determined by using a high-performance liquid chromatography (HPLC) system equipped with a pump (PU-2089 Plus; Jasco, Tokyo, Japan), an automatic injector (AS-2050 Plus), and an UV/Vis detector (UV-1575) as previously reported (Lee et al., 2016). A reverse phase C18 column (Gemini, 250 × 4.6 mm; Phenomenex, Torrance, CA) with a guard column (SecurityGuard Guard Cartridge kit, Phenomenex, Torrance, CA) was used for analyzing the CUR. For calculating the drug encapsulation efficiency, the CUR-loaded NA was dissolved in DMSO and further diluted with the mobile phase. The mobile phase was composed of acetonitrile, tetrahydrofuran, and water (35:20:45, v/v/v). The flow rate was 1 mL/min and the injection volume was 20 μL. The absorbance was monitored at 425 nm by an UV/Vis detector. The inter- and intra-day variances were within the acceptable range.

The particle characteristics of the c-Lys/CUR NA and the Lys-αTS/CUR NA were investigated. The mean diameter, polydispersity index, and the zeta potential values of the c-Lys/CUR NA and the Lys-αTS/CUR NA dispersions in DW were measured using DLS and laser Doppler methods (ELS-Z1000; Otsuka Electronics, Tokyo, Japan), according to the manufacturer’s protocol.

The morphological shapes of the c-Lys/CUR NA and the Lys-αTS/CUR NA were observed by transmission electron microscopy (TEM). An aliquot of dispersed NA was stained with 2% (w/v) phosphotungstic acid. It was loaded onto copper grids using films, dried for 10 min, and observed were by TEM (LEO 912AB OMEGA; Carl Zeiss, Oberkochen, Germany).

An aliquot of the dispersed NAs (0.15 mL), including 30 μg CUR, was introduced into Mini-GeBAflex tubes (14 kDa MWCO; Gene Bio-Application Ltd., Kfar Hanagide, Israel). They were next transferred to the release media (10 mL PBS at pH 7.4) and incubated in a shaking water bath at 50 rpm at 37 °C. Aliquots of the release media (0.2 mL) were collected at 3, 6, 24, 48, 72, and 120 h and were put into the HPLC vials. Then, equivalent volumes of the fresh release media (PBS, pH 7.4) were added. The amounts of CUR released were quantitatively determined by the described HPLC method.

### Cellular accumulation and distribution

The cellular accumulation efficiency of the CUR-loaded NA developed in this study was evaluated in MDA-MB-231 cells using flow cytometry. The MDA-MB-231 cells were purchased from the Korean Cell Line Bank (KCLB, Seoul, Korea). The cells were cultured with RPMI 1640 supplemented with 10% (v/v) FBS, 1% (v/v) penicillin (100 U/mL), and streptomycin (0.1 mg/mL) in a humidified atmosphere with 5% carbon dioxide at 37 °C. The MDA-MB-231 cells were seeded onto six-well plates at a density of 6.0 × 10^5^ cells per well and were incubated for 24 h at 37 °C. The CUR solution and the Lys-αTS/CUR NAs (corresponding to a CUR concentration of 10 μg/mL) were added to the cells and were incubated for 2 and 6 h, respectively. After washing with PBS (pH 7.4) at least thrice, the cells were collected after centrifugation at 16,100 g for 5 min. The cell pellets were resuspended with FBS solution (2%, v/v). The cellular accumulation efficiency, represented as the cell count according to the fluorescence intensity, was evaluated by a FACSCalibur Fluorescence-activated Cell Sorter (FACS^TM^) equipped with the CELLQuest software (Becton Dickinson Biosciences, San Jose, CA).

The cellular distribution of the CUR-loaded NA was assessed by confocal laser scanning microscopy (CLSM). MDA-MB-231 cells, at a density of 1.0 × 10^5^ cells per well (surface area of 1.7 cm^2^ per well), were seeded onto culture slides (BD Falcon, Bedford, MA) and were incubated for 24 h at 37 °C. The CUR solution and the Lys-αTS/CUR NA (corresponding to a CUR concentration of 10 μg/mL) were added to the cells and they were incubated for 2 and 6 h, respectively. The cells were then washed with PBS (pH 7.4) at least thrice and fixed with a 4% (v/v) solution of formaldehyde. The liquid content was removed by drying and the VECTASHIELD mounting medium with 4′,6-diamidino-2-phenylindole (DAPI) (H-1200; Vector Laboratories, Inc., Burlingame, CA) was added to the fixed cells for staining the nuclei and to prevent fading. The fluorescence signals of CUR and DAPI in the cells were observed by CLSM (LSM 880, Carl-Zeiss, Thornwood, NY).

The mechanisms of cellular entry of the Lys-αTS/CUR NA were tested by treatment with endocytosis inhibitors (genistein and chlorpromazine) in MDA-MB-231 cells (Lee et al., 2017a). The fluorescence signal of encapsulated CUR was used as an indicator of the amount of cellular uptake. The MDA-MB-231 cells were seeded onto six-well plates at a density of 4.0 × 10^5^ cells per well and were incubated for 1 day at 37 °C. Genistein (200 µM) or chlorpromazine (10 µg/mL) was co-incubated with the Lys-αTS/CUR NA (at a CUR concentration of 10 µg/mL) in MDA-MB-231 cells for 4 h. After removing the samples, the cells were washed with PBS at least thrice. The cells were detached from the bottom of the well-plate and were collected by centrifugation. The cell pellets were then suspended with PBS supplemented with FBS (2%, v/v) for flow cytometry analyses. The cell count, indicated by the fluorescence intensity, was measured using a FACS^TM^ equipped with the CellQuest software . The percentage (%) of the mean fluorescence intensity value of the (Lys-αTS/CUR NA + genistein) or the (Lys-αTS/CUR NA + chlorpromazine)-treated groups relative to that of the Lys-αTS/CUR NA-treated group was calculated.

### In vitro *anticancer activities*

The antiproliferative activity of the Lys-αTS/CUR NA was evaluated in MDA-MB-231 cells by a colorimetric assay. MDA-MB-231 cells, at a density of 5.0 × 10^3^ cells per well, were seeded onto 96-well plate and incubated at 37 °C for 24 h. Lys, Lys-αTS, CUR, and the Lys-αTS/CUR NA, corresponding to CUR concentrations of 0.5, 1, 2.5, 5, and 10 μg/mL, respectively, were treated for 48 and 72 h; αTS at concentrations of 0.1, 1, 10, 25, and 50 μg/mL was added to the cells and incubated for 72 h. After eliminating those samples, the cells were treated with the MTS-based CellTiter 96^®^ AQ_ueous_ One Solution Cell Proliferation Assay Reagent (Promega Corp., Fitchburg, WI) at 37 °C, according to the manufacturer’s protocol. The absorbance was measured at 490 nm with a multi-mode microplate reader (SpectraMax i3, Molecular Devices, Sunnyvale, CA) and the cell viability was presented by comparing with the absorbance value of the control (no treatment) group.

The apoptotic effects of the CUR-loaded NA were assessed in MDA-MB-231 cells. MDA-MB-231 cells were cultured according to the aforementioned method, seeded on six-well plates at a density of 1.0 × 10^5^ cells per well and were incubated for 24 h at 37 °C. Lys, Lys-αTS, CUR, and the Lys-αTS/CUR NA (corresponding to a CUR concentration of 2 μg/mL) was added to the cells and were incubated for 24 h. The cells were washed with PBS (pH 7.4) at least thrice and the cell pellets were collected by centrifugation at 16,100 g for 5 min. They were then suspended in the reaction buffer of the fluorescein isothiocyanate (FITC) Annexin V Apoptosis Detection Kit (BD Pharmingen, BD Biosciences, San Jose) and the cells were stained with Annexin V-FITC and propidium iodide (PI), according to the manufacturer’s protocol. The fluorescence intensity values of both the reagents in the cells were analyzed by a FACS^TM^ equipped with the CellQuest software.

### Near-infrared fluorescence (NIRF) imaging

The biodistribution of the fabricated NA in the MDA-MB-231 tumor-xenografted mouse model was assessed by a real-time NIRF imaging. Cy5.5 was conjugated to Lys-αTS as a NIRF dye. Cy5.5-NHS was conjugated to the amine group of Lys via the formation of an amide bond. Lys-αTS (20 mg) was dispersed in PBS (5 mL at pH 7.4), and the Cy5.5-NHS solution (0.2 mg in 0.02 mL DMSO) was added to that dispersion. This mixture was stirred for 4 h and dialyzed against DW with a dialysis membrane (MWCO: 6–8 kDa). It was lyophilized for further use. CUR was encapsulated in the Cy5.5-Lys-αTS NA using the dialysis method described in the Section “Fabrication and characterizations of CUR-loaded NAs”. The content of Cy5.5 in the NA was quantitatively analyzed by measuring the absorbance at 675 nm using a multi-mode microplate reader (SpectraMax i3, Molecular Devices, Sunnyvale, CA).

Female BALB/c nude mice (five weeks old, Charles River, Wilmington, MA) were used for preparing the MDA-MB-231 tumor-xenografted mouse model. The mice were reared in a light-controlled room maintained at a temperature of 22 ± 2 °C and a relative humidity of 55 ± 5%. The experimental procedures were approved by the Animal Care and Use Committee of the Kangwon National University. Aliquots of the MDA-MB-231 cell suspension (2.0 × 10^6^ cells in 0.1 mL) were injected into the dorsal regions of the mice. The tumor volume (V, mm^3^) was calculated using the Equation (1):

(1) $$\text{V}=0.\text{5}\times \text{longest diameter}\times {\left(\text{shortest diameter}\right)}^{\text{2}}$$

After the tumor volume reached 150–200 mm^3^, 0.1 mL aliquots of the Cy5.5 solution or the Cy5.5-Lys-αTS/CUR NA dispersion, containing 100 µg/kg dose of Cy5.5, were injected to the tail veins of the mice. Prior to a whole body scanning by the Spectral Lago X (Spectral Instruments Imaging; Tucsan, AZ), the mice were anesthetized by the inhalation of isoflurane (2.5%). The Cy5.5 filter (excitation: 640 nm; emission: 710 nm) was selected for the detection of NIRF in the body and the AMIView software (ver. 1.7.01) was used for image analyses. The whole body scan images were taken at 0 (pre), 4, and 24 h post-injection. The liver, lungs, heart, kidneys, spleen, and the tumor were dissected from the mice at 24 h post-injection for *ex vivo* NIRF imaging. The *ex vivo* images were obtained by using a fluorescence *in vivo* imaging system (FOBI; NeoScience Co., Ltd., Suwon, Korea) with a red laser source, according to the manufacturer’s protocol.

### In vivo *antitumor efficacies*

The *in vivo* antitumor efficacies of the developed nanosystems were assessed in MDA-MB-231 tumor-xenografted mouse models prepared with BALB/c nude mice (female, five weeks old). The mice were reared in a light-controlled room at a temperature of 22 ± 2 °C and a relative humidity of 55 ± 5%. The animal study was approved by the Animal Care and Use Committee of the Kangwon National University. The MDA-MB-231 cells were cultured according to the aforementioned protocols and aliquots of the MDA-MB-231 cell suspension (2.0 × 10^6^ cells in 0.1 mL) were injected into the dorsal regions of the mice. The tumor volume was calculated using the Equation (1). When the tumor volume was approximately 150 mm^3^, the mice were randomly divided into four groups and the tumor volume was monitored. Lys-αTS and Lys-αTS/CUR NA were dispersed in water for injection. The CUR solution (1 mg/mL) was prepared by dissolving CUR in 37.5% (w/w) PEG400 solution. Lys-αTS, CUR or the Lys-αTS/CUR NA was injected into the tail veins of the mice, at a dose of 5 mg/kg CUR, on days 5, 8, 12, 15, 19, and 22. The body weight of each mouse was monitored along with the tumor volume. On the final day, the tumor tissues were dissected and fixed in formaldehyde (4%, v/v) solution for histological staining. The tumor tissues with a thickness of 6 μm were deparaffinized and hydrated with ethanol. The tumors were stained with hematoxylin and eosin (H&E) and further processed for a terminal deoxynucleotidyl transferase dUTP nick end labeling (TUNEL) assay by standard procedures. For the TUNEL assay, 3,3′-diaminobenzidine (DAB) was added and the tissues were incubated for the development of color for the detection of DNA fragmentation produced by the apoptotic signaling cascades.

### Statistical analyses

The Student’s *t*-test was used for statistical analyses of the data. All experiments were performed at least thrice. All the data are presented as the mean ± standard deviation (SD).

## Results and discussion

### Synthesis and characterization of Lys-αTS

**Figure 1.**
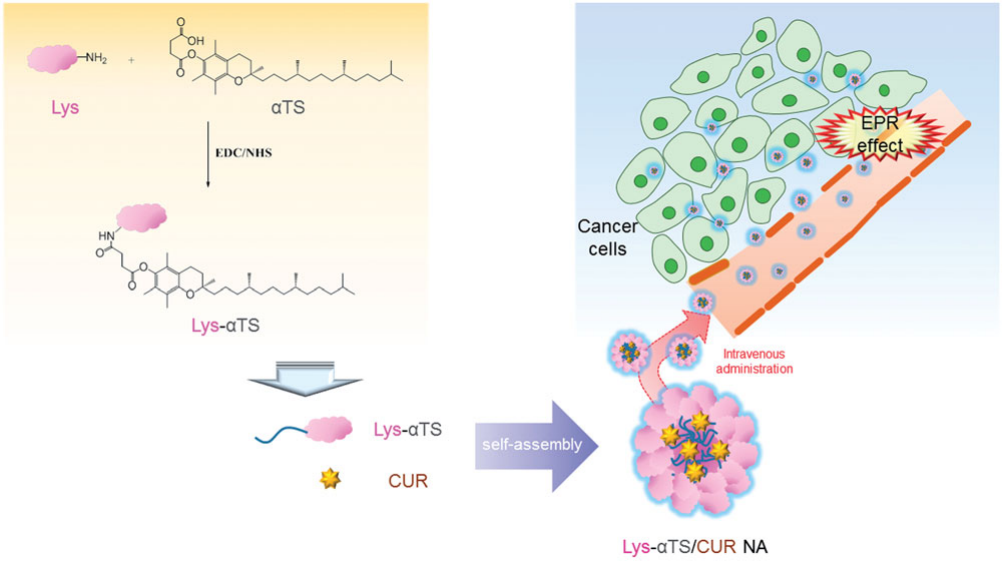
Schematic illustration of tumor-targeted therapy of Lys-αTS/CUR NA.

The CUR-loaded Lys-αTS NA was fabricated and its therapeutic potential in breast cancer was evaluated in this study (Figure 1). To prepare the structure of the Lys-based NA, αTS was used as a hydrophobic residue, which was covalently bonded to Lys. Lys has 6 lysine and 11 arginine residues and has amine groups in its structure (Masuda et al., 2005). The amine group of Lys was linked to the carboxylic acid group of αTS to produce an EDC/NHS-coupled amide bond (Figure 1). Due to the hydrophobic nature of αTS, Lys-αTS may show properties of self-assembly in aqueous environments, leading to the formation of the NA structure. It is reported that Lys also has 11 free carboxylic acid groups, although two residues (aspartate 52 and glutamic acid 35) are directly involved in the chemical reactions of Lys (Lin & Koshland, 1969). Therefore, the addition of EDC/NHS reagents in Lys could contribute to self-conjugation via the formation of an amide bond between the amine and carboxylic acid groups of Lys itself. As a control group, c-Lys was synthesized without the addition of αTS in this study. Commonly, the use of both EDC and NHS reagents might contribute to the formation of a stable amide bond between the amine and the carboxylic acid groups without side reactions.

**Figure 2.**
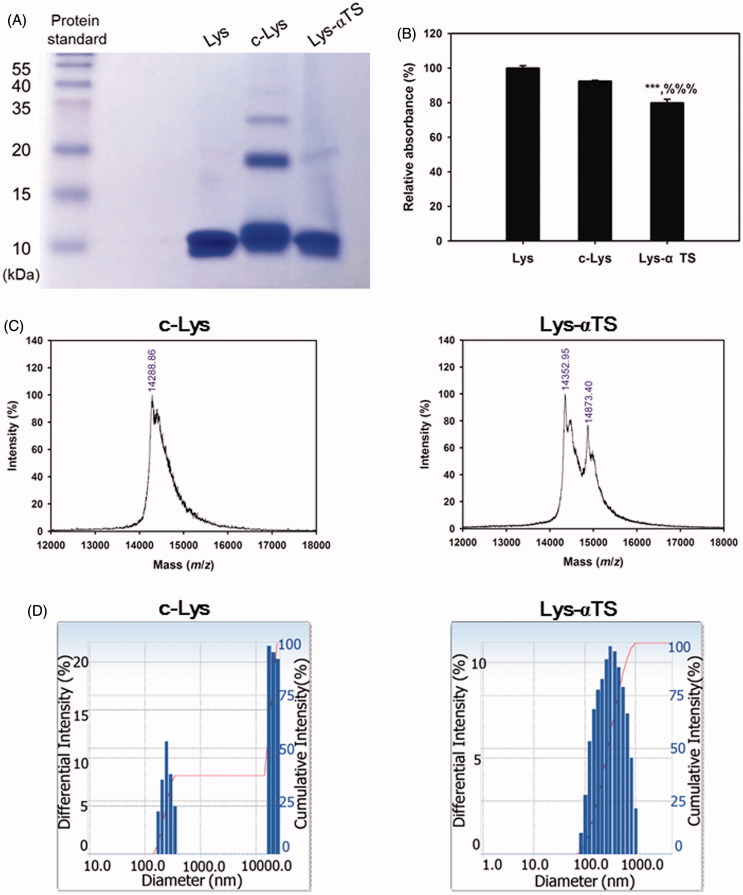
Synthesis of c-Lys and Lys-αTS and their characterizations. (A) The result of SDS-PAGE assay. Protein standard, Lys, c-Lys, and Lys-αTS were loaded onto the wells for gel electrophoresis. (B) The result of TNBS assay. Each point represents the mean ± SD (*n* = 3). ***p < .001, compared with Lys group. ^%%%^*p* < .001, compared with c-Lys group. (C) MALDI-TOF choromatograms of c-Lys and Lys-αTS. Relative intenstiy (%) accroding to mass (*m*/*z*) is plotted. (D) Size distribution diagrams of c-Lys and Lys-αTS. Differential intensity according to the diameter is plotted.

The synthesis of c-Lys and Lys-αTS was verified by several experimental methods (Figure 2; Supplementary Figures S1 and S2). The change in the amino acid sequence, corresponding to the molecular weights of the synthesized Lys derivatives, was tested by a SDS PAGE assay (Figure 2(A)). In the Lys lane, the band corresponding to the monomer was primarily observed, while bands corresponding to dimers and multimers were hardly seen. However, the c-Lys group exhibited multiple bands, indicating the presence of monomers, dimers, and trimers. Contrary to the c-Lys group, the Lys-αTS group showed the formation of a smaller number of multimers, which was similar to the Lys group. During the process of Lys-αTS synthesis, αTS seemed to have successfully conjugated to Lys without the formation of self-crosslinks within the structure of Lys. The secondary structure of Lys was investigated by CD analysis (Supplementary Figure S1). In the far UV region, the minimum peak was formed around 208 nm in this study as reported (Cho et al., 2011). It is known that minimum peaks at 208 and 222 nm imply the contribution of *n* → π* transfer to the peptide bond of α-helices (Price, 2000; Cho et al., 2011). The CD values (at 208 nm) of c-Lys and Lys-αTS were significantly lower than that of Lys. The formation of crosslinks within the structure of Lys and the attachment of αTS to Lys seemed to alter the secondary structure of Lys, including a reduction in the α-helix content. The tertiary structure of Lys was studied by fluorescence intensity detection (Supplementary Figure S2). It is known that an emission spectrum in the range of 330–345 nm may be due to the tryptophan residues in proteins (Zemser et al., 1994). The highest fluorescence intensity of Lys determined in this study was observed at an excitation wavelength of 341 nm (Supplementary Figure S2). The relative fluorescence intensity values of c-Lys and Lys-αTS at the tested excitation and emission wavelengths were 86.7 and 59.1% respectively, compared to that of Lys. The crosslinking of Lys itself and the Lys-αTS conjugation seemed to alter the tertiary structure of Lys.

The chemical conjugation of αTS to Lys was also tested by using the TNBS assay (Figure 2(B)). The TNBS assay has been used for the quantitative analysis of the amine groups. The relative absorbance value of Lys-αTS was significantly lower than those of Lys and c-Lys (*p* < .001). From the relative absorbance values, approximately 20% of the amine groups in Lys seemed to participate in the reaction with the carboxylic acid groups of αTS and those of Lys itself. The lower number of amine groups quantified in Lys-αTS than in c-Lys, indicated the efficient covalent bonding of αTS to Lys in Lys-αTS compared with self-crosslinking of Lys in c-Lys. The molecular weights of the synthesized materials were determined by MALDI-TOF analysis (Figure 2(C)). The major peak of Lys was seen at an *m*/*z* value of 14325.77 and the presence of multimers were also observed in this study (data not shown; Farmer & Caprioli, 1991). In case of the c-Lys group, the major peak was observed at *m*/*z* 14288.86 and the low intensity peaks of multimers were also observed (data not shown). Two major peaks at *m*/*z* values of 14352.95 and 14873.40 were present in the spectrum of Lys-αTS. While the peak at *m*/*z* 14352.95 may indicate Lys itself, the peak at *m*/*z* 14873.40 seems to be related to Lys-αTS. The particle characteristics of c-Lys and the Lys-αTS dispersion in aqueous media were also evaluated (Figure 2(D) and Supplementary Table S1). The dispersion of the c-Lys group exhibited a mean diameter of 833 nm and a polydispersity index of 0.49. Aggregates were observed in the aqueous environment as shown in the particle size distribution diagram (Figure 2(D)). In comparison with these data, the Lys-αTS dispersion group showed a mean diameter of 286 nm and a polydispersity index of 0.24. A unimodal peak was observed in the size distribution profile of the Lys-αTS group. The attachment of a hydrophobic moiety (αTS in this study) can produce smaller particles with narrower size distributions, than the self-crosslinking of Lys (as observed in the c-Lys group).

### Preparation and characterization of Lys-αTS/CUR NA

**Figure 3.**
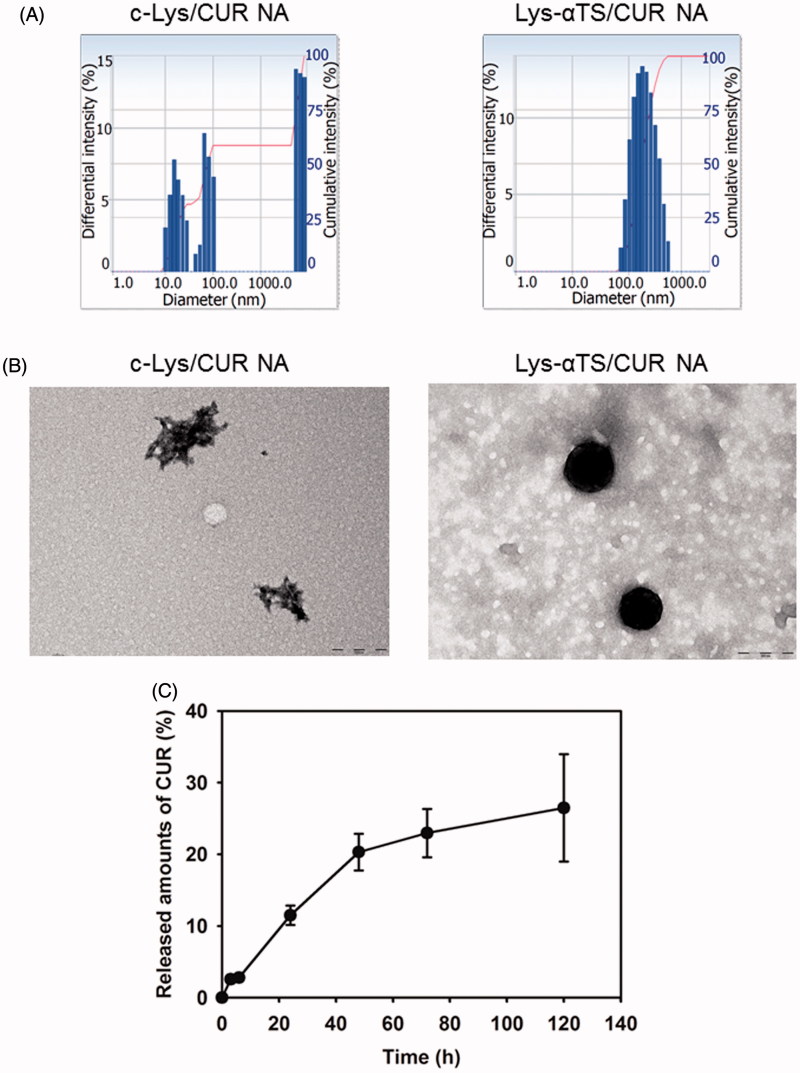
Particle characterizations of c-Lys/CUR NA and Lys-αTS/CUR NA. (A) Size distribution diagrams of c-Lys/CUR NA and Lys-αTS/CUR NA dispersion. The differential intensity is plotted according to the mean diameter. (B) TEM images of c-Lys/CUR NA and Lys-αTS/CUR NA dispersion. The length of the scale bar is 200 nm. (C) Drug release profile of Lys-αTS/CUR NA. Released amounts of CUR (%) from Lys-αTS/CUR NA dispersion are presented. Each point represents the mean ± SD (*n* = 3).

The CUR-loaded NAs were prepared and their particle properties were evaluated (Figure 3 and Supplementary Table S1). Upon loading the CUR onto the c-Lys NA and the Lys-αTS NA, their hydrodynamic size and polydispersity index were reduced. It indicated that more compact nanocarriers could be formed upon drug loading. The zeta potential values of both the c-Lys/CUR NA and the Lys-αTS/CUR NA were positive. The drug encapsulation efficiency values of the c-Lys/CUR NA and the Lys-αTS/CUR NA were 70 and 64%, respectively. However, the hydrodynamic size of the c-Lys/CUR NA (493 ± 82 nm) was higher than that of the Lys-αTS/CUR NA (213 ± 18 nm). In case of the c-Lys/CUR NA group, the mean diameter seemed to be less suitable for EPR effect (aimed at passive tumor targeting) rather than the Lys-αTS/CUR NA group, and the formation of aggregates was also observed (Figure 3(A); Bae & Park, 2011). Moreover, according to the TEM images, the c-Lys/CUR NA exhibited a fibril-like shape while the Lys-αTS/CUR NA showed a round morphology (Figure 3(B)). It is known that the hen egg white Lys can easily form amyloid fibrils after treatment with several factors (such as heat, salt, and organic solvents; Arnaudov & de Vries, 2005; Ow & Dunstan, 2013). The formation of irreversible fibrillary protein aggregates and their accumulation in the body may lead to amyloidosis, similar to Alzheimer’s and prion diseases (Arnaudov & de Vries, 2005). The short fibril aggregate shape together with the higher hydrodynamic size and the broad size distribution of the c-Lys/CUR NA seemed to be unsuitable for its *in vivo* application. Thus, only the Lys-αTS/CUR NA group was further tested in the following studies.

The release pattern of CUR from the Lys-αTS/CUR NA was tested at conditions of normal physiological pH (Figure 3(C)). The released amounts of CUR from the Lys-αTS/CUR NA on days 1 and 5 were 11.5 ± 1.4 and 26.5 ± 7.5%, respectively. The initial burst release (initial 24 h in this study) was not so severe and a sustained drug release was observed for 120 h at a pH of 7.4. Sustained drug release has been regarded as one of the ideal characteristics of injection formulations, as it can reduce the frequency of doses and enhance patient compliance. It is expected that the observed sustained drug release from the Lys-αTS/CUR NA may contribute to its improved antitumor efficacy.

### Cellular accumulation and distribution

**Figure 4.**
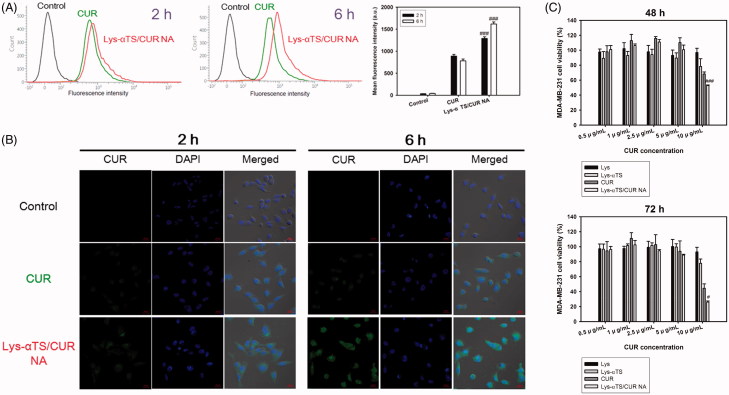
Cellular uptake and antiproliferation studies in MDA-MB-231 cells. (A) Cellular accumulated amounts of CUR quantitatively analyzed by flow cytometry. CUR or Lys-αTS/CUR NA (10 μg/mL CUR concentration) was incubated for 2 and 6 h. Black, green, and red colors indicate control, CUR, and Lys-αTS/CUR NA group, respectively. Each point represents the mean ± SD (*n* = 3). ###*p* < .001, compared with CUR group. (B) Intracellular distribution of NPs monitored by CLSM imaging. CUR or Lys-αTS/CUR NA (10 μg/mL CUR concentration) was incubated for 2 and 6 h. Green and blue colors indicate CUR and DAPI, respectively. The length of the scale bar in the image is 20 μm. (C) Antiproliferation assay in MDA-MB-231 cells. Cell viability (%) values of Lys, Lys-αTS, CUR, and Lys-αTS/CUR NA, according to CUR concentrations, are presented after 48 and 72 h incubation. Each point represents the mean ± SD (*n* = 3). #*p* < .05, compared with CUR group. ###*p* < .001, compared with CUR group.

The cellular accumulation and intracellular distribution of the developed NA were studied in MDA-MB-231 cells by flow cytometry and CLSM imaging analyses, respectively (Figures 4(A,B)). MDA-MB-231 cells are human breast adenocarcinoma cell lines that are used for the evaluation of the anticancer activities of nanocarriers targeting breast cancers (Jeong et al., 2017; Lee et al., 2017b).

The cellular accumulation efficiency of the Lys-αTS/CUR NA was evaluated in MDA-MB-231 cells (Figure 4(A)). In this study, the intrinsic fluorescence signal of CUR was used for detecting the cellular movement of the developed NAs in cancer cells. The mean fluorescence intensity values of the Lys-αTS/CUR NA at 2 and 6 h were 1.45- and 2.07-folds higher than those of the CUR group, respectively (*p* < .001). The mean fluorescence intensity of the Lys-αTS/CUR NA group at 6 h was 25.4% higher than the value at 2 h. On the contrary, the mean fluorescence intensity of CUR at 6 h was slightly reduced compared to the value at 2 h. A similar pattern was also observed in the results of CLSM imaging (Figure 4(B)). A strong intracellular fluorescence signal was observed for the Lys-αTS/CUR NA group rather than for the CUR group after 2 and 6 h of incubation. The Lys-αTS/CUR NA showed a higher cellular accumulation efficiency than that of the CUR solution, which may be due to the mechanism of endocytosis of nanocarriers. It seems that the endocytosis of nanocarriers can increase the cellular entry rate of the drug cargo rather than the passive diffusion of the drug itself. Moreover, the electrostatic interaction between the positive zeta potential of the Lys-αTS/CUR NA and the negative charge of the cellular membrane might also contribute to its enhanced cellular uptake.

The mechanisms of endocytosis of the developed Lys-αTS/CUR NA were investigated by flow cytometry analysis after treatment with endocytosis inhibitors (Supplementary Figure S3). Genistein or chlorpromazine has been used to elucidate the mechanisms of caveolae- or clathrin-mediated endocytosis (Lee et al., 2017a). In particular, the percentages of fluorescence intensity of the (Lys-αTS/CUR NA + genistein) and (Lys-αTS/CUR NA + chlorpromazine) groups were 94.55 ± 2.29 (*p* < .05) and 67.05 ± 1.94% (*p* < .001), respectively, relative to that of the Lys-αTS/CUR NA group. Co-treatment of chlorpromazine and Lys-αTS/CUR NA significantly reduced the cellular uptake amount of CUR (*p* < .001). It is possible to conclude that clathrin-mediated endocytosis might be the principal route for endocytosis of the developed Lys-αTS/CUR NA in MDA-MB-231 cells.

### In vitro *anticancer activities*

The *in vitro* anticancer activities of the Lys-αTS/CUR NAs were demonstrated in MDA-MB-231 cells (Figure 4(C); Supplementary Figures S4 and S5). The antiproliferative efficacies of Lys, Lys-αTS, CUR, and Lys-αTS/CUR NA were compared by MTS-based assays (Figure 4(C)). Lys did not show severe cytotoxicity in the tested CUR concentration range. Lys also exhibits negligible cytotoxicity in breast cancer cell lines (such as MCF-7; Mahanta et al., 2015). The half maximal inhibitory concentration (IC_50_) value of αTS after 72 h of incubation in MDA-MB-231 cells was 25.73 ± 1.19 μg/mL (Supplementary Figure S4). The anticancer activities of αTS, mediated through apoptosis and chemosensitivity, is already reported (Kanai et al., 2010). Due to the presence of αTS in Lys-αTS, Lys-αTS also exhibited slight antiproliferative effects. However, its antiproliferative efficacy was lower than those of CUR and the Lys-αTS/CUR NA. The Lys-αTS, CUR and the Lys-αTS/CUR groups exhibited higher cytotoxicity on longer periods of incubation. Notably, after 72 h of incubation, the difference between the IC_50_ values of CUR (10.33 ± 0.67 μg/mL) and Lys-αTS/CUR NA (7.95 ± 0.09 μg/mL) was significant (*p* < .01) (Supplementary Table S2). In both the incubation groups (48 and 72 h of incubation), the cell viability of the Lys-αTS/CUR NA was significantly lower than that of CUR at a CUR concentration of 10 μg/mL (*p* < .001 and .05, respectively). The antiproliferative efficacy of the Lys-αTS/CUR NA was more improved than CUR, which can be explained by the higher cellular accumulation efficiency of the Lys-αTS/CUR NA in spite of the differences in various details of the experimental conditions (such as the incubation time and the CUR concentration) as shown in Figures 4(A,B). It seems that using a nanosized carrier can increase the endocytosis of CUR in cancer cells, subsequently leading to the enhanced antiproliferative efficiency.

An apoptosis assay was performed to reveal the mechanisms of anticancer activities of the developed NAs (Figure S5). It is reported that CUR can induce apoptosis in MDA-MB-231 cells and subsequently contribute to cancer cell death (Wang et al., 2016). Especially, αTS may have additional proapoptotic activities in cancer cells via mitochondrial inhibition and the production of superoxide radicals (Gruber et al., 2014; Qu et al., 2016). The apoptotic effects in the Lys, Lys-αTS, CUR, and the Lys-αTS/CUR NA-treated groups in MDA-MB-231 cells were assessed by Annexin V-FITC and the PI staining method (Supplementary Figure S5). Lower right (LR; Annexin V-FITC positive and PI negative) and upper right (UR; Annexin V-FITC positive and PI positive) panels in the Supplementary Figure S5 indicate early apoptosis and late apoptosis/cell death, respectively. Therefore, the sum of population percentages in (LR + UR) panels was used to evaluate the apoptotic effects in this study. Notably, the Lys-αTS/CUR NA group exhibited a higher percentage of apoptotic populations in (LR + UR) panels than the CUR group. The more efficient induction of apoptosis in the Lys-αTS/CUR NA group may be due to its higher cellular accumulation efficiency than that in the CUR group. From the results of *in vitro* anticancer activities, the Lys-αTS NA can be used efficiently for the delivery of CUR to cancer cells.

### NIRF imaging

**Figure 5.**
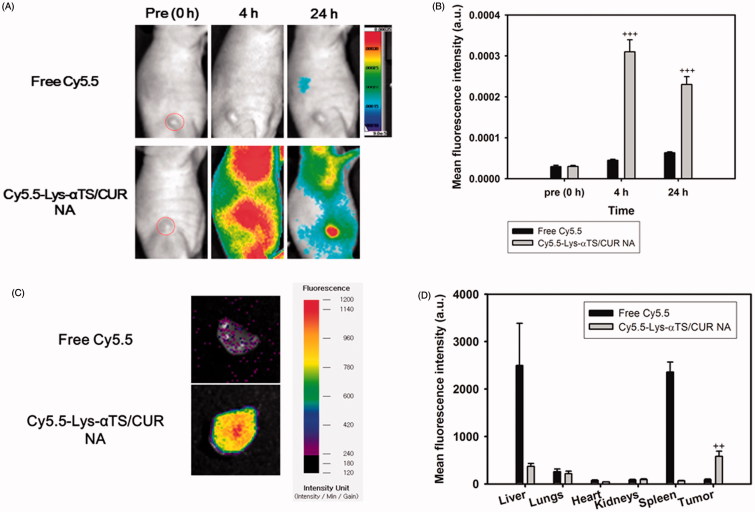
NIRF imaging test in MDA-MB-231 tumor-bearing mouse model. Free Cy5.5 or Cy5.5-conjugated Lys-αTS/CUR NA was injected into the tail vein of the mouse model. (A) Whole body-scanned images acquired at 0 (pre), 4, and 24 h post-injection. Red dashed circle indicates the tumor region. (B) Mean fluorescence intensity values in the tumor region. Each point represents the mean ± SD (*n* = 3). +++*p* < .001, compared with free Cy5.5 group. (C) *Ex vivo* NIRF image of dissected tumor tissues at 24 h post-injection. (D) Mean fluorescence intensity values in liver, lungs, heart, kidneys, spleen, and tumor are presented. Each point represents the mean ± SD (*n* = 3). ++*p* < .01, compared with free Cy5.5 group.

Tumor targetability of the fabricated Lys-αTS/CUR NA was verified by NIRF imaging in MDA-MB-231 tumor-xenografted mouse models (Figure 5). Cy5.5-NHS was conjugated as a NIRF dye to the amine group of Lys via an amide bond. As shown in Figure 5(A), the Cy5.5-Lys-αTS/CUR NA was distributed in the body while free Cy5.5 was hardly observed in the body due to its immediate excretion at 4 h. Notably, the Cy5.5-Lys-αTS/CUR NA mainly accumulated in the tumor region at 24 h post-injection. According to the quantitative data analysis at 24 h (Figure 5(B)), the mean fluorescence intensity of the Cy5.5-Lys-αTS/CUR NA in the tumor region was 3.63-fold higher than that of the free Cy5.5 (*p* < .001). This was further confirmed by *ex vivo* NIRF imaging studies with the dissected tumor tissues (Figure 5(C)). A strong fluorescence signal was detected in the Cy5.5-Lys-αTS/CUR NA group in comparison to the free Cy5.5 group. The biodistribution of the injected Cy5.5-Lys-αTS/CUR NA was also evaluated by *ex vivo* NIRF imaging (Figure 5(D)). The fluorescence intensity values in the liver, lungs, heart, kidneys, spleen, and tumor were compared in the free Cy5.5 and the Cy5.5-Lys-αTS/CUR NA-treated groups. The fluorescence intensity in the tumor tissue of the Cy5.5-Lys-αTS/CUR NA-treated group was 6.67-fold higher than the free Cy5.5-treated group (*p* < .01). Foreign materials are prone to be trapped in the reticuloendothelial system (RES), such as the liver and spleen, via phagocytosis. Avoiding the accumulation of nanoparticles in RES organs and a selective delivery to the tumor region have been considered as common objectives for the development of tumor-targeted nanosystems of anticancer agents. The relative fluorescence intensity ratios of tumor to (liver + spleen) in the free Cy5.5 and the Cy5.5-Lys-αTS/CUR NA groups were 0.018 and 1.321, respectively. The values imply a higher tumor selectivity of the Lys-αTS/CUR NA after its intravenous injection. The observed *in vivo* tumor targetability of the Lys-αTS/CUR NA may reduce the unwanted effects of CUR and improve its anticancer activities.

### In vivo *anticancer activities*

**Figure 6.**
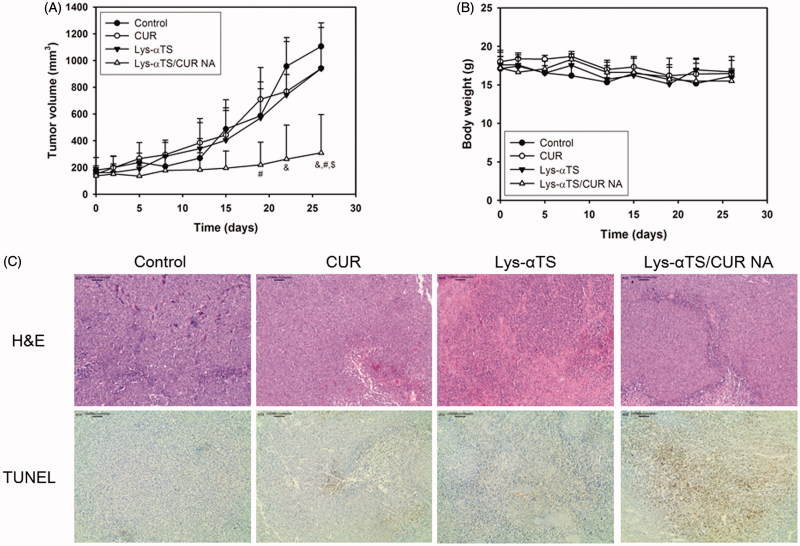
*In vivo* anticancer activity tests in MDA-MB-231 tumor-bearing mouse models. (A) Tumor volume profiles of control, CUR, Lys-αTS, and Lys-αTS/CUR NA groups. CUR, Lys-αTS and Lys-αTS/CUR NA were injected intravenously on day 5, 8, 12, 15, 19, and 22, respectively. Each point indicates the mean ± SD (*n* ≥ 3). &*p* < .05, compared with the control group. #*p* < .05, compared with CUR group. $*p* < .05, compared with Lys-αTS group. (B) Profiles of body weight of control, CUR, Lys-αTS, and Lys-αTS/CUR NA groups. Each point indicates the mean ± SD (*n* ≥ 3). (C) Staining images of dissected tumor tissues. The microscopic images of H&E staining (upper panel) and TUNEL assay (lower panel) are shown. The length of scale bar in the image is 100 μm.

The *in vivo* anticancer activities of the Lys-αTS/CUR NA were tested in MDA-MB-231 tumor-bearing mouse models after multiple dosing (Figure 6). For CUR delivery to the tumor region, the introduction of nanocarriers is necessary to overcome the drawbacks (that is, poor water-solubility). Following the *in vivo* and *ex vivo* biodistribution studies (Figure 5), the *in vivo* anticancer activities were verified by studying the inhibition of tumor growth, changes in body weight, and immunohistological staining of dissected tumor tissues. As shown in Figure 6(A), among all experimental groups, the highest inhibition of tumor growth was observed in the Lys-αTS/CUR NA group. It is noted that the tumor volume of the Lys-αTS/CUR NA group was significantly lower than those of all the other groups on the final day (*p* < .05). The tumor volume on day 26 of the Lys-αTS/CUR NA group was 28.0, 32.8, and 32.9% of those of the control, CUR, and the Lys-αTS groups, respectively. Also, there was no significant alteration in the body weight during the entire monitoring period (Figure 6(B)). It may imply the absence of severe systemic toxicity after the intravenous administration of the developed CUR-loaded NA. The dissected tumor tissues from each group were examined by H&E and TUNEL staining (Figure 6(C)). In particular, the brown color in the staining results of TUNEL assay, indicative of apoptotic events in tumor tissues, of the Lys-αTS/CUR NA group, was stronger than those of all the other groups. As observed in the results of the cellular apoptosis assay (Supplementary Figure S5), apoptosis seemed to be efficiently induced in the tumor region after the arrival of the CUR-loaded NA. The most efficient inhibition of tumor growth by the Lys-αTS/CUR NA is supported by this apoptotic efficacy. Conclusively, the αTS-linked enzymatic nanocarrier can selectively deliver CUR to the tumor tissue, so that it can exert its efficient *in vivo* anticancer activities.

## Conclusions

The Lys-αTS/CUR NA was developed as an enzymatic nanosystem for the imaging and therapy of breast cancers. Lys-αTS was synthesized by the formation of an amide bond between the amine group of Lys and the carboxylic acid group of αTS. CUR, as a poorly water-soluble drug, was incorporated into the Lys-αTS nanostructure. Compared with the c-Lys/CUR NA, Lys-αTS/CUR NA exhibited a smaller hydrodynamic size (213 nm mean diameter), a narrower size distribution, and a more spherical shape, which can be regarded as a safe and efficient tumor-targeting drug delivery system. The sustained drug release from the Lys-αTS/CUR NA was also observed for five days at normal physiological conditions. The cellular accumulation, antiproliferative effects, and the apoptotic efficiencies of the Lys-αTS/CUR NA were significantly higher than those of CUR. According to the results of the NIRF imaging test in MDA-MB-231 tumor-bearing mouse models, the Lys-αTS/CUR NA showed a selective accumulation in the tumor, rather than in the other organs and tissues. Following intravenous injections in the MDA-MB-231 tumor-xenografted mouse models, the Lys-αTS/CUR NA exhibited a higher inhibition of tumor growth and apoptotic events in the tumor tissue than the other groups. All these findings indicate that the Lys-αTS/CUR NA can be used as efficient and safe enzyme-based nanocarrier for the therapy of breast cancers.

## Supplementary Material

IDRD_Cho_et_al_Supplemental_Content.docx
