# Supplementary material for: An α-tocopheryl succinate enzyme-based nanoassembly for cancer imaging and therapy
Source: Drug Deliv. 2018 Mar 8;25(1):738–49. doi: 10.1080/10717544.2018.1446476 (PMC6058571; doi:10.1080/10717544.2018.1446476)
Supplement: IDRD_Cho_et_al_Supplemental_Content.docx [file IDRD_A_1446476_SM8923.docx]

**Supplementary Information**

**An α-tocopherol succinate enzyme-based nanoassembly for cancer imaging and therapy**

Song Yi Lee and Hyun-Jong Cho^*^

*College of Pharmacy, Kangwon National University, Chuncheon, Gangwon 24341, Republic of Korea*

^*^Corresponding authors. Tel.: +82 33 250 6916; fax: +82 33 259 5631.

*E-mail address*: hjcho@kangwon.ac.kr (H.-J. Cho).


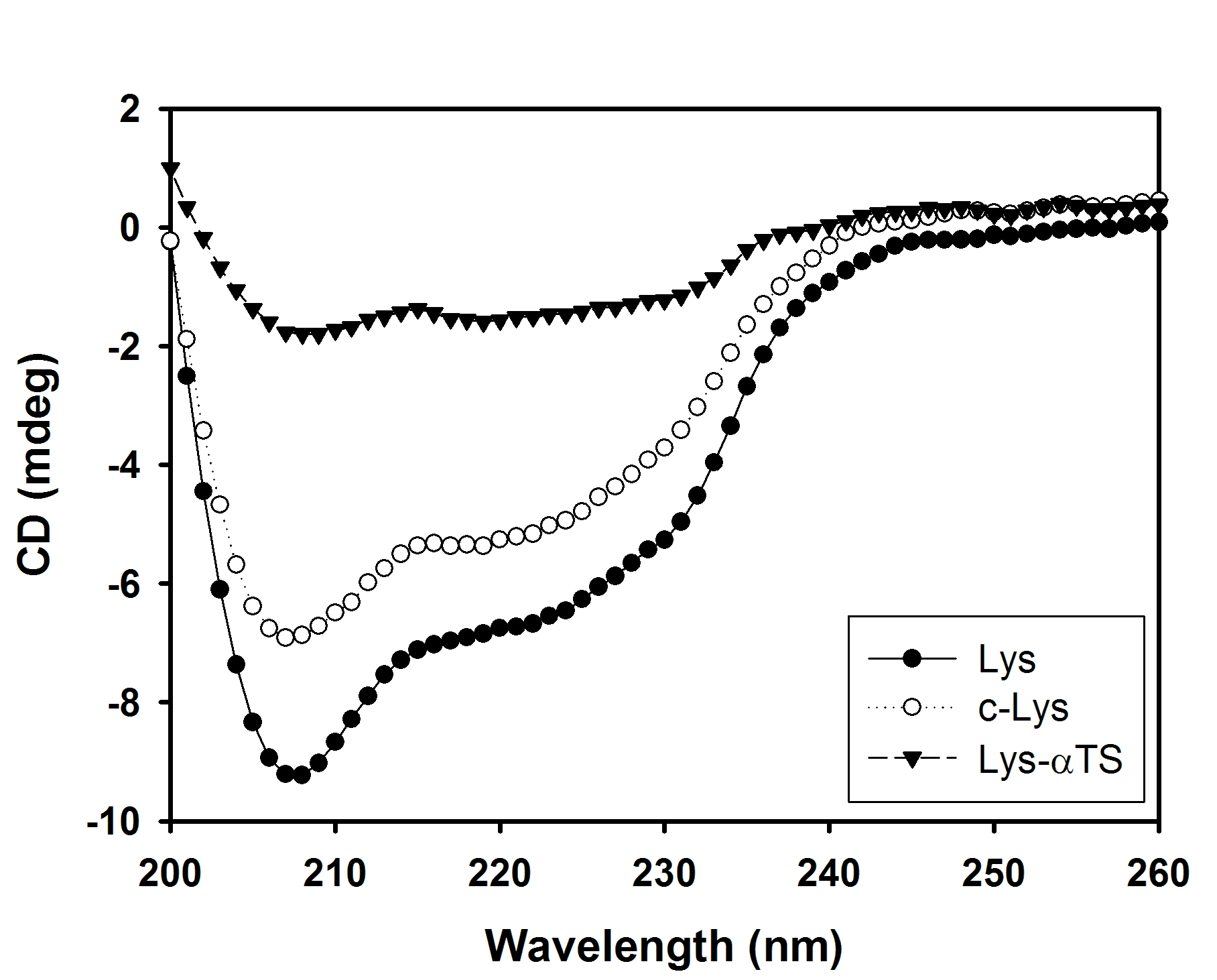


**Fig. S1.** Circular dichroism data of Lys, c-Lys, and Lys-αTS. CD values according to the wavelength are plotted.


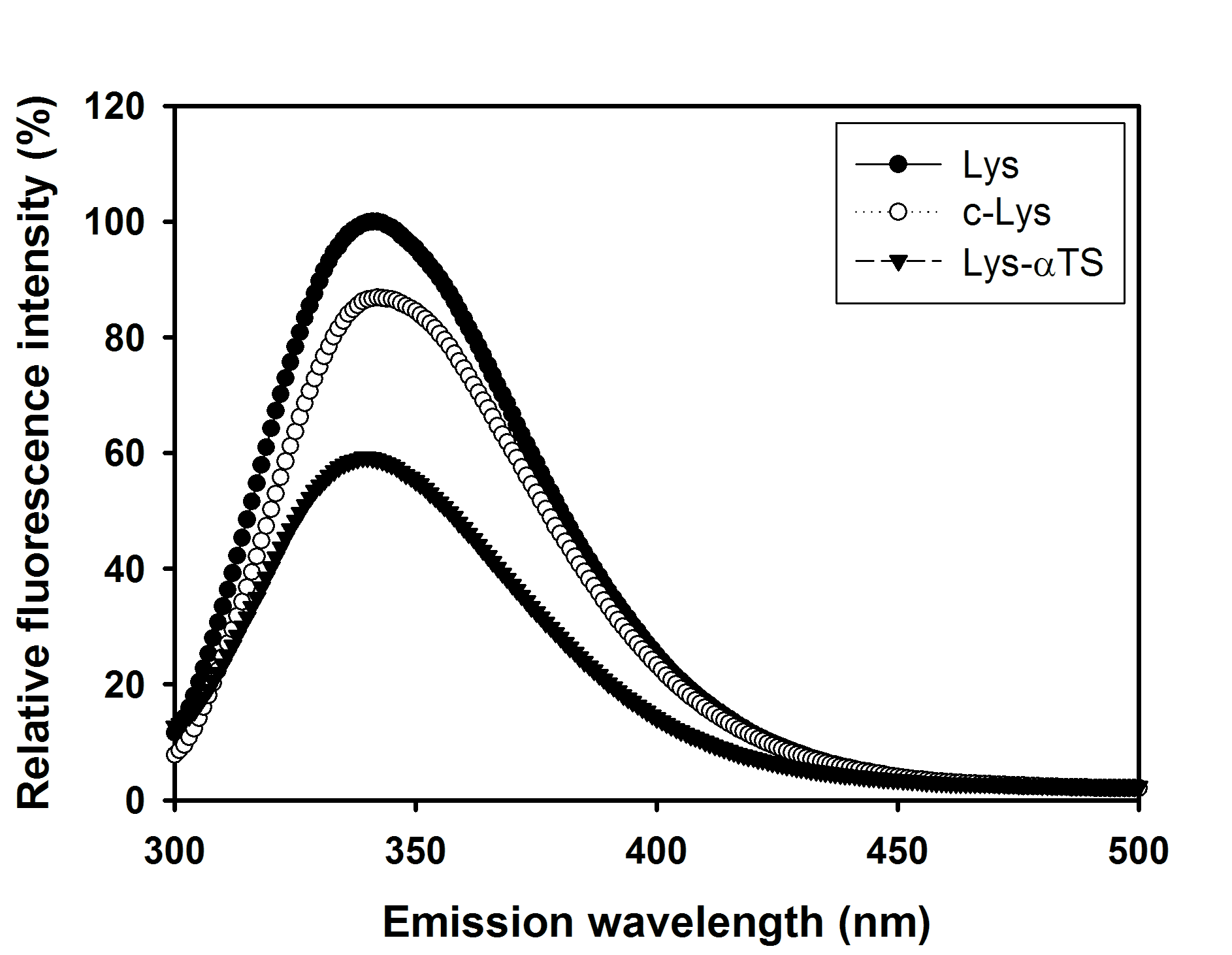


**Fig. S2.** Fluorescence intensity profiles of Lys, c-Lys, and Lys-αTS. The emission spectra of Lys, c-Lys, and Lys-αTS are shown. The relative fluorescence intensity (%) of each group, compared to that of Lys group, is plotted according to the emission wavelength.


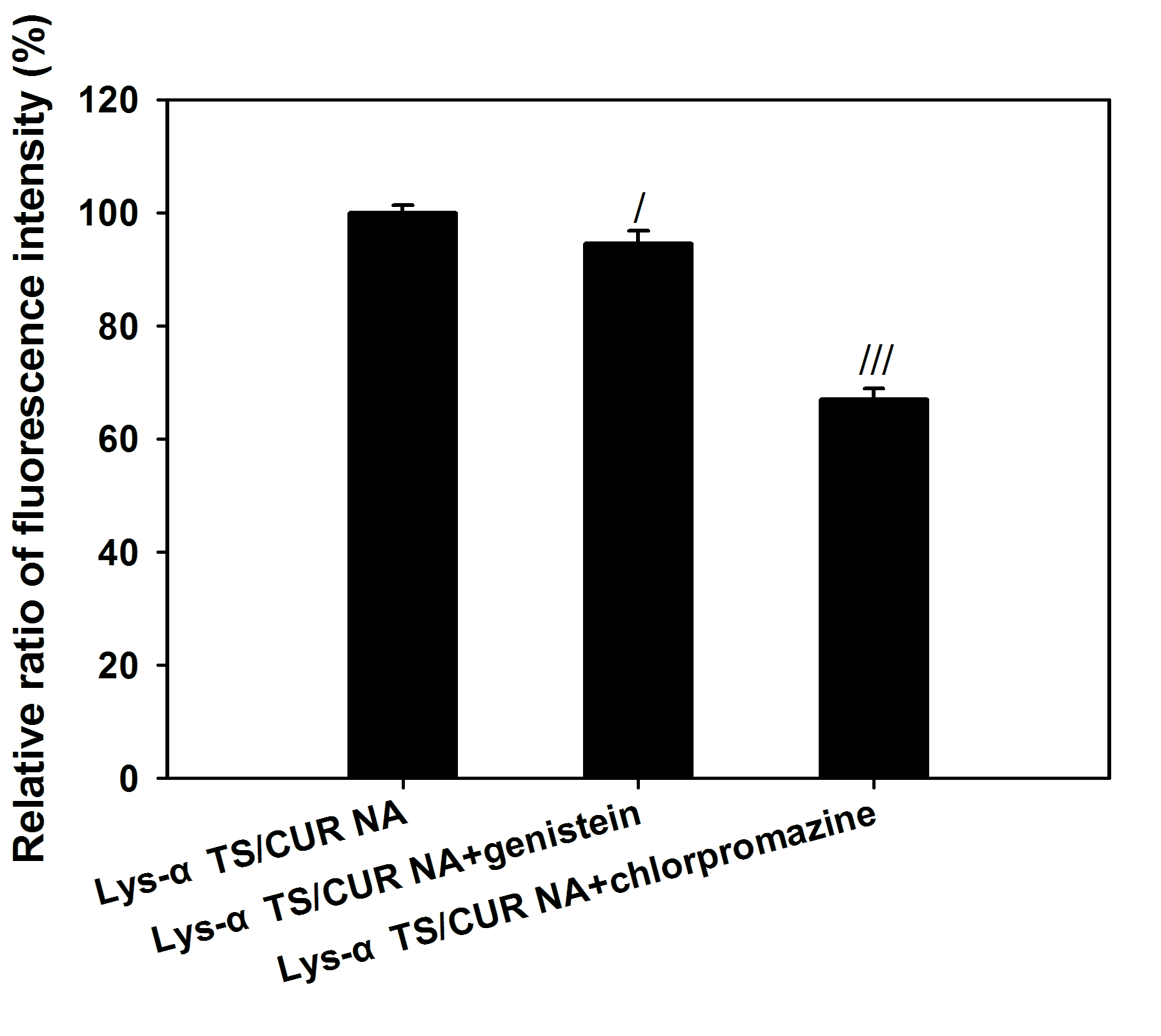


**Fig. S3.** Cellular uptake mechanism of developed CUR-loaded NA. Endocytosis inhibitors (genistein and chlorpromazine) were co-incubated with Lys-αTS/CUR NA for 4 h. Relative ratio of fluorescence intensity of each group, compared to that of Lys-αTS/CUR NA group, is shown. Each point represents the mean ± SD (*n* = 3). ^/^*p* < 0.05, compared with Lys-αTS/CUR NA group. ^///^*p* < 0.001, compared with Lys-αTS/CUR NA group.


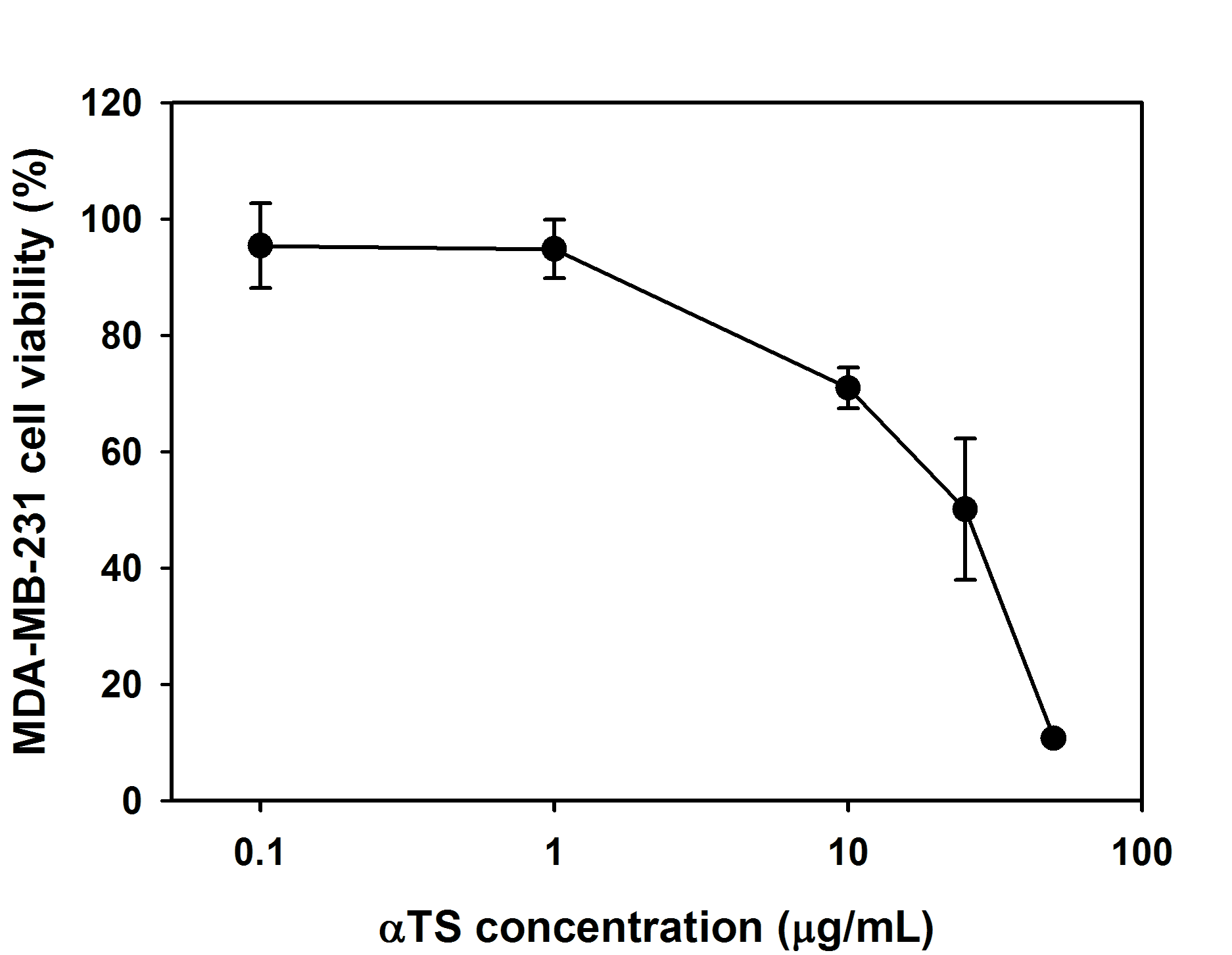


**Fig. S4.** Antiproliferation efficacy of αTS in MDA-MB-231 cells. Cell viability (%) was measured by MTS-based assay. Each point represents the mean ± SD (*n* = 3).


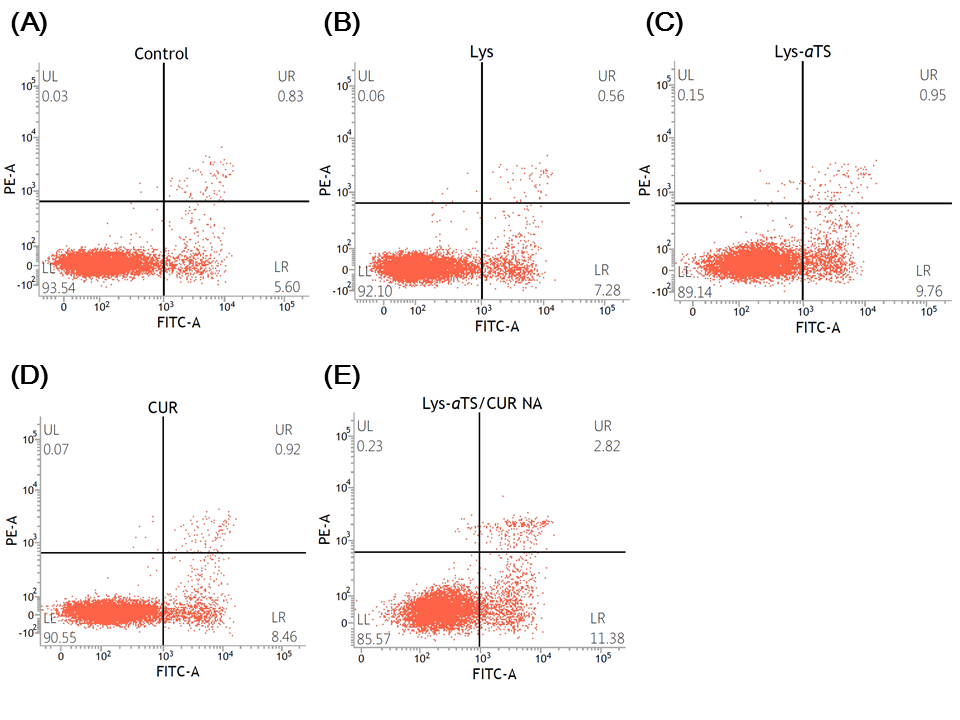


**Fig. S5.** Apoptosis assay in MDA-MB-231 cells. Lys, Lys-αTS, CUR, and Lys-αTS/CUR NA were incubated and the apoptotic events were measured by Annexin V-FITC and PI-based assay. FITC-A and PE-A indicate the fluorescence signal of Annexin V-FITC and PI, respectively. UL, LL, LR, and UR indicate upper left, lower left, lower right, and upper right panel, respectively. Cell population percentage (%) is shown in each panel.

**Table S1.** Particle characterizations of developed formulations.

| **Formulation** | **Mean diameter**  **(nm)** | **Polydispersity index** | **Zeta potential (mV)** | **Encapsulation efficiency**  **(%)** |
| --- | --- | --- | --- | --- |
| c-Lys | 833 ± 142 | 0.49 ± 0.08 | 42.88 ± 0.81 | ‒ |
| Lys-αTS | 286 ± 2 | 0.24 ± 0.01 | 36.20 ± 1.61 | ‒ |
| c-Lys/CUR NA | 493 ± 82 | 0.32 ± 0.05 | 42.23 ± 5.72 | 70.2 ± 0.9 |
| Lys-αTS/CUR NA | 213 ± 18 | 0.23 ± 0.07 | 34.93 ± 1.43 | 63.5 ± 13.1 |

Each sample was dispersed in DW.

$$\mathrm{Encapsulation} \mathrm{efficiency} (\%)=\frac{\mathrm{actual} \mathrm{amount} \mathrm{of} \mathrm{CUR} \mathrm{in} \mathrm{NA}}{\mathrm{input} \mathrm{amount} \mathrm{of} \mathrm{CUR} \mathrm{in} \mathrm{NA}}\times100$$

Data are presented as the mean ± SD (*n* ≥ 3).

**Table S2.** IC_50_ values of CUR and CUR-loaded NA in MDA-MB-231 cells.

| **Formulation** | **IC50 value (μg/mL)** | |
| --- | --- | --- |
|  | **48 h** | **72 h** |
| CUR | 15.2 ± 0.6 | 10.3 ± 0.7 |
| Lys-αTS/CUR NA | 15.5 ± 1.4 | 8.0 ± 0.1^##^ |

Data are presented as the mean ± SD (*n* = 3).

^##^*p* < 0.01, compared with CUR group.
